# What constitutes responsiveness of physicians: A qualitative study in rural Bangladesh

**DOI:** 10.1371/journal.pone.0189962

**Published:** 2017-12-18

**Authors:** Taufique Joarder, Asha George, Syed Masud Ahmed, Sabina Faiz Rashid, Malabika Sarker

**Affiliations:** 1 BRAC James P Grant School of Public Health, BRAC University, Dhaka, Bangladesh; 2 Department of International Health (Health Systems), Johns Hopkins Bloomberg School of Public Health, Baltimore, Maryland, United States of America; 3 School of Public Health, University of Western Cape, Cape Town, South Africa; La Trobe University, AUSTRALIA

## Abstract

Responsiveness entails the social actions by health providers to meet the legitimate expectations of patients. It plays a critical role in ensuring continuity and effectiveness of care within people centered health systems. Given the lack of contextualized research on responsiveness, we qualitatively explored the perceptions of outpatient users and providers regarding what constitute responsiveness in rural Bangladesh. An exploratory study was undertaken in Chuadanga, a southwestern Bangladeshi District, involving in-depth interviews of physicians (n = 17) and users (n = 7), focus group discussions with users (n = 4), and observations of patient provider interactions (three weeks). Analysis was guided by a conceptual framework of responsiveness, which includes friendliness, respecting, informing and guiding, gaining trust and optimizing benefits. In terms of *friendliness*, patients expected physicians to greet them before starting consultations; even though physicians considered this unusual. Patients also expected physicians to hold social talks during consultations, which was uncommon. With regards to *respect* patients expected physicians to refrain from disrespecting them in various ways; but also by showing respect explicitly. Patients also had expectations related to *informing and guiding*: they desired explanation on at least the diagnosis, seriousness of illness, treatment and preventive steps. In *gaining trust*, patients expected that physicians would refrain from illegal or unethical activities related to patients, e.g., demanding money against free services, bringing patients in own private clinics by brokers (*dalals*), colluding with diagnostic centers, accepting gifts from pharmaceutical representatives. In terms of *optimizing benefits*: patients expected that physicians should be financially sensitive and consider individual need of patients. There were multiple dimensions of responsiveness- for some, stakeholders had a consensus; context was an important factor to understand them. This being an exploratory study, further research is recommended to validate the nuances of the findings. It can be a guideline for responsiveness practices, and a tipping point for future research.

## Introduction

The concept of responsiveness is derived from the fields of medical ethics, human rights, and human development [1]. It was first used in the context of human resources for health (HRH) by the Joint Learning Initiative on Human Resources for Health, without substantial elaboration [2]. The World Health Report 2006 signaled the importance of ‘responsiveness’ in HRH by including it as a key element of performance [3]. In this paper, drawing on the definition of health systems responsiveness [4], HRH responsiveness is defined as the ‘social actions by health providers to meet the legitimate expectations of patients’.

Responsiveness of health workers is important as a right in of its own, while also being instrumental to supporting care seeking by patients. Studies show that poor responsiveness may dissuade patients from early care seeking, diminish their interest in adopting preventive health information [5–7], and decrease their trust in health service providers [8]. Literature also indicates that discourteous behavior from physicians often inhibits care-seeking by the elderly, patients suffering from non-communicable diseases [9], expectant and new mothers [10], and the lesbian-gay-bisexual-transgender (LGBT) community [11–13], leading to compromised wellbeing.

Responsiveness is also critical to the specific context of Bangladesh. According to three surveys, carried out in 1999, 2000, and 2003, the most important predictor of satisfaction of patients with health providers was the behavior of the providers (expressed in the form of respect and politeness) with the patients, rather than their clinical competence [14–16]. Dissatisfaction among service seekers with the provider’s behavior has often been expressed in the form of physical violence, according to both recent media reports [17–19] and scientific journal articles [20–22]. Physicians sometimes responded to these violent acts by engaging in strikes and refusing to provide services [23–25]. These tensions, many of which result from the lack of HRH responsiveness, can lead to patient suffering and even death [17,26]. A growing number of social science studies conducted in Bangladesh are corroborating media reports, denoting the humiliation of the patients by providers [26–28]. These incidents call for a rigorous exploration of the underlying issues, such as responsiveness of physicians.

The government of Bangladesh has recognized the importance of responsiveness and announced its commitment to develop a responsive health workforce [29]. The government of Bangladesh exercises its stewardship role in health sector through a sector-wide approach known as the Health, Population and Nutrition Sector Development Program (HPNSDP) [30]. The HPNSDP expressed its intent to improve the responsiveness of Bangladeshi HRH and also set service providers’ quality criteria, which includes “interpersonal relations and responsiveness of the service providers” [31]. Bangladesh’s commitment for a responsive health workforce is also highlighted in the 2011 National Health Policy [32].

There are few studies on the responsiveness of HRH [33–36], especially on physicians; and none in Bangladesh. Among these studies, one focused primarily on HRH performance and responsiveness was discussed as a component but was not explored in detail, either conceptually or methodologically [34]. Another study involved telephone interviews in eight European countries, inquiring about patients’ views on health care in their respective countries, with a focus on patients’ involvement in care and choice of health care provider [33]. This quantitative study reported on doctor-patient communication, involvement in treatment decisions, and choice of provider, but it neither mentioned the source of variables used, nor reported any formative study on responsiveness constructs. Another study was done in Brazil on nursing staff, which described the psychometric steps in developing an instrument to assess nursing care responsiveness but lacked conceptual clarification on how the scale items had been derived [36]. Finally, a study of physicians from Thailand employed simulated patient method to analyze the degree of responsiveness (along with patient-centeredness, therapeutic decision, and cost) in terms of opening hours, waiting time, consultation time, requests for follow-up visits, and politeness of physician [35]. This study did not explore qualitatively what responsiveness meant to service seekers or providers or what its constituent elements were.

Since there is lack of a clear understanding in existing literature regarding responsiveness of physicians, this study aimed to fill out this knowledge gap and contribute to this vital, yet ignored aspect of performance of HRH. This study explored qualitatively the perceptions and practices of outpatient healthcare users and providers (physicians) regarding the elements of responsiveness of physicians in rural Bangladesh. This study was carried out as formative qualitative research for a larger study that aimed to derive items for a scale to measure responsiveness of physicians in rural Bangladesh.

## Materials and methods

### Conceptual framework

**Fig 1:**
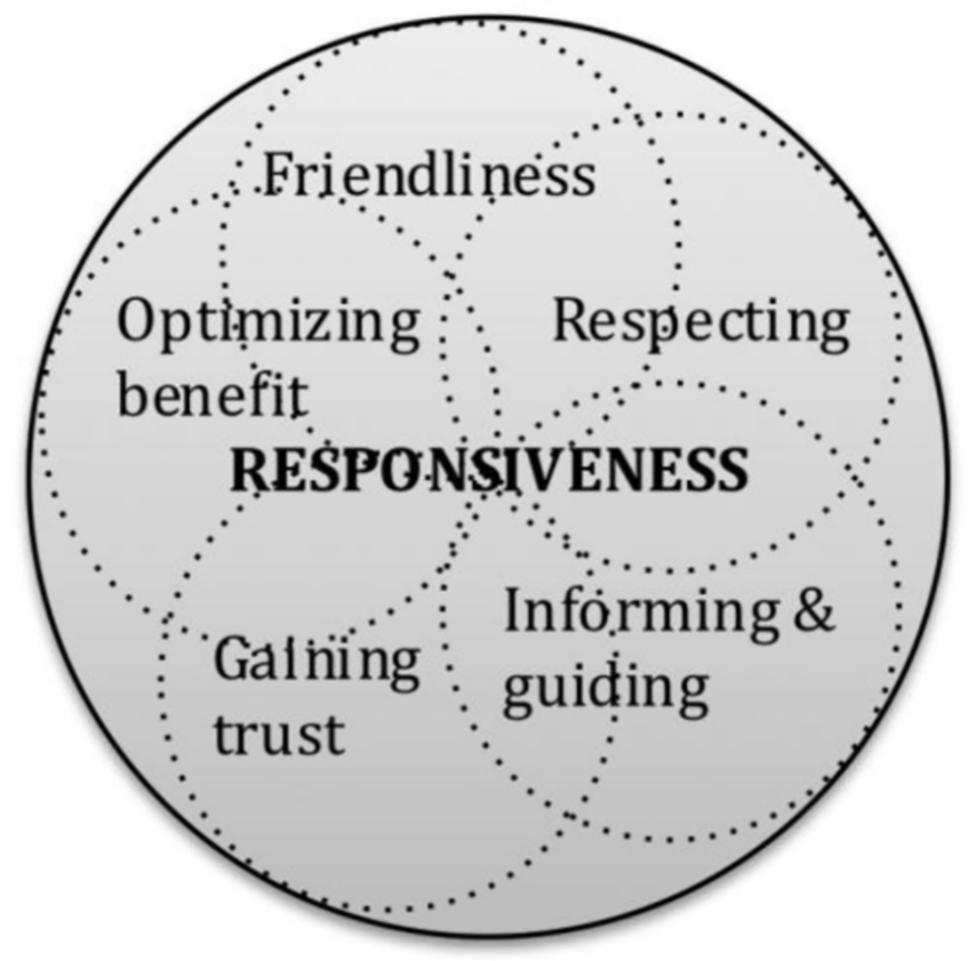
Domains of responsiveness of physicians. List of reviewed literature and the detailed process of deriving these domains are described elsewhere [38]. These domains are interlinked, and the components may often overlap.

Based on literature review (primarily studies on health systems responsiveness [4,5,7,37], with supplements from studies on HRH responsiveness [33–36,38], doctor-patient communication/relationship [39], quality of care [14,28], and patient satisfaction [14,27]) and inputs from the experts based at schools of public health in Baltimore (Johns Hopkins University) and Dhaka (BRAC University), the following five domains of HRH responsiveness were adopted for this study (Fig 1):

1. **Friendliness:** How a provider shows friendly demeanor to a patient.
2. **Respecting:** How a provider explicitly shows respect to a patient.
3. **Informing and guiding:** How a physician gives information about health condition and guides a patient.
4. **Gaining trust:** How a physician gains the trust of a patient, or refrains from doing something that breaches trust.
5. **Optimizing benefits:** How a physician tries to optimize the benefits of a patient, going beyond the consultation.

### Study design

This was exploratory qualitative research at the micro-level of health systems (i.e., considering the role of individuals in health provision and utilization) analysis [40]. Multiple data sources and methods were used to triangulate and validate the findings. This study involved in-depth interviews (IDI) with 17 physicians who worked in the public (seven), private (five) and informal (five) sectors; IDIs with seven service users; focus group discussions (FGD) with service users (two sessions each with males and females); and observations in public (one week in Upazila Health Complex [UpHC]), private (one week in a for-profit clinic and an NGO-clinic), and informal (consultation chamber-cum-medicine shop of a village doctor) sector settings (Table 1).

**Table 1:** Characteristics of respondents and observation settings.

**In-depth interview with public sector physicians**		
Number	7	
Gender	2 females and 5 males	
Range of graduation year	1982–2009	
**In-depth interview with private sector physicians**		
Number	5 (2 of them retired from public sector, 1 was accepted in public sector and waiting to join, and only 2 had no linkage with public sector)	
Gender	1 female and 4 males	
Range of graduation year	1973–2013	
**In-depth interview with village doctors**		
Number	5	
Gender	5 males	
Range of number of years in practice	2–32	
Range of level of formal education (excluding training in medicine)	secondary–bachelor	
**In-depth Interview with service users**		
Number	7	
Gender	4 females and 3 males	
Range of age in years	25–48 (females: 25–45; males: 45–48)	
Range of level of education	primary–masters (females: primary-honors; males: honors-masters)	
Types of occupation	females: homemaker, kindergarten teacher and high school teachers; males: high school teachers, businessmen	
**FGD with service users**		
Number of sessions	4 (2 with females, 2 with males)	
Number of participants	7–8 in each session	
Range of age in years	19–72 (females: 19–59; males: 31–72)	
Range of level of education	primary–masters (females: primary-masters; males: primary-honors)	
Type of occupation	females: college and high school teachers and custodial staff; males: college and school teacher, retired government official, businessman, farmer	
**Observations**		
Setting		3 settings: public sector (consultation rooms in an UpHC), private sector (consultation rooms in a for-profit private clinic and an NGO-clinic), and informal sector (consultation rooms of 3 village doctors in a village bazaar)
Duration		1 week in each setting

### Study population and sampling

This qualitative research was conducted in the southwestern part of Bangladesh, in all three rural Upazilas (Alamdanga, Damurhuda, and Jibannagar) of the Chuadanga District. One of the reasons for choosing Chuadanga was personal; as it was the main data collector and first author’s ancestral District. This allowed a better understanding of the local language, culture, society, geography, and politics. Secondly, Chuadanga shares a fair similarity with the rest of the country in terms of ethno-cultural and socio-demographic profiles. The three Upazilas also share similar socio-demographic characteristics, are located close to one another geographically (highest distance of only 52 kilometers by road, between Alamdanga and Jibannagar), and are not known for any particular difference.

Study participants were selected based on the principles of heterogeneous purposive sampling [41] and observation locations on both purposive and convenience sampling.

For IDIs of service users and male FGDs, respondents had to be older than 18 years, who went to a physician at least twice in their lifetime, with the last visit within the last year, while for female FGD groups, respondents were drawn from two local educational institutions, as they could not agree on a common time and place to meet otherwise.

### Data collection

The location and time of data collection was determined based on the preference of the respondents. All IDIs and FGDs were interviewed using pre-tested interview guides and were digitally recorded with the permission of the respondent. Duration of the IDIs ranged from 35 minutes to one hour 15 minutes and that of FGDs from 45 minutes to one hour 30 minutes. Data were collected using thematic topic guides, covering issues related to socio-demographic characteristics, general questions (detailed description of a consultation process that s/he encountered, general expectations from physicians, etc.), specific questions on prompted aspects of responsiveness (responsiveness elements extracted from literature review), and finishing questions (agreement, disagreement, suggestions). In each location the first author spent seven days: the first day for gaining a grasp on the surroundings and people; second and third day for observing overall functions and relationships; and the remaining four days for focused observation of consultations with particular attention to responsiveness elements identified through literature review as well as IDIs and FGDs. A topic guide was used for observation too.

### Data analysis

Since the IDIs and FGDs were digitally recorded, two Research Assistants (RA) transcribed them verbatim. The first author listened to each record, checked the transcripts line-by-line, transcribed himself if anything was found missing, and finalized the transcripts. He also word-processed the observation notes.

The analysis process included the following steps: familiarizing with the data, developing coding schema or framework, coding, grouping, and interpreting the data. Initial codes were derived from literature as *a priori* codes; inductive codes were added during data familiarization, and finally the codes were applied to text segments. Codes were ultimately grouped into the following five themes: 1) Friendliness (combining clear communication and inter-personal aspects of care); 2) Respecting (combining dignity and confidentiality); 3) Informing and guiding (combining autonomy, participation, and considerate care); 4) Gaining trust (combining attention and appearance); and 5) Optimizing benefits (from beneficence to patients). In order to increase validity, TJ and MS independently coded the dataset. SMA was involved where a third opinion was warranted to reach consensus or resolve controversial issues.

### Ethical considerations

We obtained ethical approval from the Ethical Review Board of BRAC University, Dhaka, Bangladesh. In addition we obtained permission from Directorate General of Health Services, Bangladesh Private Medical Practitioners Association, and the head of respective institutions (UpHCs and private clinics), where observations were made. Informed written consent was obtained from participants. A local advisory committee based in Dhaka was formed to tackle any unforeseen ethical issues identified during data collection.

## Results

### Description of respondents and observation settings

All seven public-sector physicians practiced privately, as dual practice is allowed in Bangladesh; however, we requested they respond based only on their experiences and views pertaining to public-sector work. Among the five respondents from the private sector, only two never worked in the public sector; others however had some involvements with public sector: either retired from public service, or would start public service shortly. Male service user IDI respondents were older and more educated than the female sample. Female FGD respondents belonged to a higher education group than the general populace, owing to their being sampled from two educational institutions. Observation in the public sector continued from eight in the morning to two in the afternoon, while observation in the private sector setting took place during the physician's working hours. Observation of village doctors continued from half past eight in the morning until eight in the night.

### Domains of responsiveness

This section is organized along the five domains of responsiveness of physicians. Elements of each domain, as found through data analysis, are summarized in Table 2. Perspectives of stakeholders along with observations on important elements of physicians’ responsiveness are discussed below.

**Table 2:** Elements of responsiveness of physicians in each domain.

Name of Domain	Components of Domain
**Friendliness**	Greeting, identifying self by the physician, engaging in social talk, showing friendliness, giving reassurance, not using jargon or professional language, not showing hierarchical difference, positive non-verbal communications, being humorous, holding closing conversation.
**Respecting**	Expressing respect, listening to complaints completely and attentively, taking consent, allowing patients to ask questions, being culturally sensitive, refraining from discriminations (based on socio economic status, gender, religion, type of disease, or any other consideration), avoiding interruptions during consultation, having an acceptable appearance, and establishing or maintaining discipline inside consultation room.
**Informing and guiding**	Communicating limitations, helping patients to find the right physician, explaining to patients different aspects of their disease or condition (cause, diagnosis, prognosis, treatment, preventive aspects, side effects of drugs, and result of tests), involving patients in decision making and care, providing patients with information on health promotion and disease prevention, writing prescription legibly, and facilitating follow up.
**Gaining trust**	Maintaining confidentiality of information, referring immediately if necessary, taking help from colleagues in confusion, gaining trust, being service-oriented not businesslike, and refraining from illegal or unethical activities.
**Optimizing benefits**	Counseling on social or family issues if related to the disease, going for a home visitation if demanded, considering individual need of the patient while prescribing, facilitating utilization of local resources, and showing financial sensitivity.

#### Friendliness

An ideal greeting, according to patients, includes giving *salam* (Muslim way of saying hello), introducing him/herself, asking about the patient’s wellbeing, addressing the patient appropriately (mother or father to elderly; brother, sister, sister-in-law or *bhabi* to similar aged persons; *babu*, *shona*, etc. to children), and asking them to take a seat. Physicians acknowledged the importance of greetings as they read about this in their textbooks, but they perceived this to be out of local custom:

*"Actually there is no custom of saying 'hi*, *hello' in our culture*. *Sometimes we just ask them to come in*, *and upon entering they simply start telling their problems*.*"* [IDI with a public sector physician, female, year of graduation 2009]

#### Observations revealed it was usually the patient who initiated the greeting

According to patients, physicians were also expected to engage in social talk. In particular, patients valued providers asking about the patient’s family. Additional topics also included the patient's profession, education, that day's weather, etc. A retired public sector physician, who at the time of data collection was involved in private practice, said that he commonly exchanged smiles and engaged in social talks with his elderly patients, but engaging in social talks, in general, was not the norm. In observations we found, private sector physicians seemed focused on the administrative practice of writing the patient's name, age, and gender for prescription purposes, rather than for conversation. In the public sector, the name was already written on the ticket, so they did not ask at all. We found engaging in social talks by formal sector physicians in general very uncommon, in contrast to village doctors who were quite adept at this.

Patients said that they wanted their physicians to display some friendly gestures, such as remembering the face or name of the patient from a previous encounter, calling the patient by the name in a friendly tone, asking or making comment about an event of the patient's family, praising the patient (about clothing or anything else), or asking for an opinion of the patient about anything (weather, politics, etc.).

Patients expect physicians to be encouraging and reassuring so that patients do not have to worry or be frightened of the therapeutic procedures. A patient said, "Half of the disease is cured only by reassurance." [IDI with a teacher, female, 40 years]

According to patients, reassurance can be expressed both verbally and non-verbally. Reassurance-expressing speech and behavior may include phrases like ‘you have no problem’, ‘I would be able to cure your disease *inshallah* [by the grace of Allah]’, and gestures like putting hands on the shoulder of the patient, giving them courage by holding their hand, and giving courage by putting hand on the body. Almost all of the physicians acknowledged the importance of giving reassurance, which was supported by observations too. In observations of a private clinic, the physician we observed, patted gently on the shoulder of almost all the patients, and said, “It will be cured”.

It was commonly expected by interviewed patients that the physician’s behavior would not show the hierarchical difference. However, in observations there were several nonverbal cues indicating the physician’s supposed superiority: physicians sitting on a cushioned chair with a comfortable backrest and headrest with a towel hanging on the backrest, signifying their superiority, while patients sat on a small tool; physicians wearing shoes while patients were restricted from wearing them inside the consultation room; and the overall gestures of the physicians. This difference was more clearly observable in the private sector due to the very nice chairs used by the physicians. Such symbols of hierarchical differences were less pronounced, or completely absent, in consultation settings of the village doctors.

Another expectation, as expressed by the patients, was that physicians would have some sense of humor or maintain a smile during the consultation. Some physicians, both males and females, were observed to be quite humorous. One such example of being humorous is when a young lady asked for vitamin syrup, the physician replied with a humorous tone, "*leave these baby foods; you are a nice young lady now*, *not a baby anymore*."

We found closing conversation to be more common than greetings (Table 3). Typical closing conversations included shaking hands, saying *walaikum-assalam* (Muslim way of saying goodbye) and phrases like 'stay well', etc.

**Table 3:** Crosscutting themes across five domains of responsiveness of physicians.

Common themes	Friendliness	Respecting	Informing and guiding	Gaining trust	Optimizing benefits
**Gaps between physicians’ and patients’ perceptions and practices regarding responsiveness**	• Patients expected greeting words, social talks, and non-hierarchical arrangements during consultations • Physicians perceived greetings to be out of norm and had hierarchical sitting arrangements.	• Patients expressed expectation of not being discriminated, and uninterrupted consultations. • Physicians answered patients’ questions briefly, got irritated at ‘irrelevant’ questions; discriminated based on familiarity, socio-economic status, political position and power.	• Patients expected physicians to explain different aspects of the disease, and write prescription clearly and legibly. • Physicians gave insufficient explanation, and prescribed with illegible handwriting.	• Patients expected physicians would consult with others in confusion, be care-oriented and not businesslike, and refrain from illegal or questionable activities. • Physicians diagnosed or prescribed wrongly, acted businesslike by demanding fees from poor patients, suggesting specific diagnostic centers for tests, etc.; and were involved in unethical practices such as allowing *dalal*’s around consultation rooms (public sector) or reaping benefits from them (private sector).	• Patients expected home-visitation by physicians, and financial sensitivity and information regarding treatment costs. • Physicians, except the very senior ones, never went for home-visitations; and considered informing the treatment costs to be out of the scopes of consultation.
**Importance of context in understanding responsiveness**	Exchange of closing conversations was more common than introductory greetings.	Seeking consent was considered redundant by both physicians and patients, except in some specific circumstances.	Allowing autonomy may be harmful if ‘ignorant and superstitious’ patients want to consult a ‘quack’. Patients felt more comfortable relying on expert decisions by physicians, rather than shared decisions. Some physicians felt providing explanation was useless for patients, who lacked knowledge.	Patients were reportedly more satisfied with their information shared with different agencies.	Patients expected from physicians to consider their financial status before prescribing.
**Aspects of responsiveness where physicians lived up to the expectations of service seekers**	Most physicians provided some degree of reassurance, and demonstrated sense of humor (mainly in private sector).	Physicians were respectful in general; listened to patients attentively; and did not discriminate on gender, religion, disease condition, and age.	Physicians usually told patients about disease prevention and health promotion aspects related to the particular disease.	Physicians in the private sector referred patients readily if found to be non-treatable in the existing setup. Public-sector physicians did the opposite, considering the cost of referral and fearing this to be a burden for the predominantly poor patients visiting them.	A physician showed his sensitivity about cases of violence against women, and expressed his readiness to comply with the legal prerogatives of such cases. Physicians demonstrated financial sensitivity by explicitly trying to understand patients’ financial ability to undergo treatment, and even helped within their limited means.

#### Respecting

Patients mentioned they expect the physicians to show some explicit signs of respect. Disrespectful demeanor, according to patients, includes the following: using offensive words, bargaining on fees, denying treatment, stopping the patient in the middle of giving disease history, talking in an authoritative tone, scolding, and ejecting the patient from the room. On the contrary, a physician may demonstrate respect by giving honor to an elderly patient by standing up, helping her/him to sit down, and talking softly with the patient. Most of the patients admitted that physicians, in general, were not disrespectful, but there were some exceptions. The most common misconduct of physicians was condescending behavior, as reported by many patients in both IDIs and FGDs, when they said, "*Do you understand better than me*? *Then why didn't you become a doctor yourself*?” In interviews with physicians, almost all of them admitted that showing respect was necessary and they all practiced it; however, three physicians admitted they often breached it. One young public sector physician, on condition of not recording his statement, mentioned the following (notes were taken instead of a recording):

*"Many doctors scold the patients and behave rudely*. *Sometimes patients come with dirty clothes due to low socio-economic conditions; they are scolded and shamed for this too*. *I myself sometimes cannot control my temperament*. *I scolded patients severely on some occasions and ejected them out of the room*.*"* [IDI with a public-sector physician, male, year of graduation 2007]

In observation, however, we did not see any physician disrespecting a patient (Table 3); but explicitly displaying respect was uncommon too.

Patients expected that physicians would start writing the prescription only after listening to the complaints in detail and completely. Contrary to common preconceptions, we observed the physicians, both in public and private sectors, listening attentively to patients. This might be due to the fact that they spent little time on history taking, physical examination, and diagnostic tests. So they entirely depended on what the patient had to say as their chief complaints.

Regarding seeking consent, many patients considered this unnecessary except in some specific cases such as: placing the stethoscope on the chest of a female patient by a male physician, uncovering any covered part of the body or touching a part of the body while examining (except touching the forehead for fever), and examining the private parts of any patient. Physicians expressed their cognizance of the value of consent from textbooks, but in a Bangladeshi context, they also considered it redundant (Table 3).

Patients said they expected the physician to give them the opportunity to ask questions, listen to their questions attentively, encourage them to ask questions, respond to the question himself (i.e., they would not direct their assistants to answer patients), and remain patient and tactful if asked irrelevant questions. According to patients, behaviors that might denote discouragement to ask questions may be: repeatedly looking at the clock, reminding the patient to be concise, writing prescriptions while answering the question, ultra-seriousness, answering very shortly (in one word). On the other hand, actions denoting encouragement to questions may be: smiling during the consultation and answering, listening carefully and patiently to the questions, shaking head while listening, looking at the patient, and some interest-revealing words (e.g., well, hmm, etc.). In interviews with physicians, they also acknowledged the importance of answering the patient's questions. However, some physicians blamed patients for asking irrelevant questions, too many questions, or repeating the same question by persons accompanying the patient. One physician said:

*"Suppose I consulted a child patient*. *I explain the prescription to the accompanying mother*. *After some time you will see child's father coming*, *so I explain again*. *Then you will see child's grandmother coming with questions*. *These make us irritated and we often lose our nerves*.*"* [IDI with a public sector physician, male, year of graduation 2004]

Physicians admitted that they did not particularly encourage their patients through their gestures or verbal cues to ask questions. In observations, it was found the physicians answered patients' questions very briefly, even in a single word. Village doctors were observed to be quite lenient about patients' questions. In the interviews too, all of them emphatically said they would never lose patience with patients' questions, whatever they asked.

When probed on cultural sensitivity, patients demanded that physicians should prescribe treatment considering the religious and cultural orientation of the patients. Examples given by them of cultural sensitivity included: making adjustments while giving medicine to a Muslim patient during *Ramadan* (the month of fasting), suggesting '*pottho*' (diet) that is available during that season, not giving advice of doing or eating anything which is religiously prohibited, and giving ideas about the disease and treatment by using household language.

In regards to discrimination, patients said physicians usually did not discriminate based on gender, religion, disease condition, and age, but they often did on the social and political status and familiarity with the patient (Table 3). One elderly male FGD participant said:

*"Now that Awami League (ruling government party at the time of data collection) is in power*, *even if some young boys of their party go to a doctor*, *the doctor will see them first*. *I*, *being an old man*, *will have to keep waiting along with other suffering patients*.*"* [FGD participant, male]

In an interview with physicians, a female public-sector physician suggested that patients also understood the helplessness of the physicians in this regard. In this type of situations, she suggested, physicians should inform the patient and ask for permission as a gesture of courtesy and respect.

#### Informing and guiding

Patients said they often feel that they need some suggestions or assistance in finding the appropriate physician for them. They said it would be useful for them to receive suggestions regarding this from nearby physicians free of cost. Some patients demanded similar services from physicians in finding an appropriate specialist, such as a cardiologist, a nephrologist, etc.

When asked about their views on the right of the patients to go to a provider of their choice, and the physician's role in helping them in this regard, a physician brought an interesting issue. He said, in the context of Bangladesh where many patients were uneducated and ignorance or superstitions were prevalent, allowing patients to go to the provider of their choice might turn out to be harmful. So, he said, he would not mind if the patient wanted to go to a qualified formal sector provider, but if he wanted to visit a 'quack' (the term used to mean village doctors), then it would not be acceptable (Table 3).

Explanation was one of the most important things that a patient desired from a physician. Patients, according to both IDIs and FGDs with them, expect that the physician would explain everything to them, such as cause of the disease, diagnosis (at least the name of the disease), prognosis and severity, treatment (at least explain the prescription), side effects of the medicines (if any), report of diagnostic tests (if any), and preventive measures of disease (especially the diet). Many physicians in Bangladesh did not find enough time to explain things to their patient. So, they delegated this to their assistants and untrained pharmacists. Patients expected that the physician himself would explain everything to them. It is also important that the physicians make sure patients have understood the explanation, as many patients are illiterate or are simply unfamiliar with the basic anatomy and physiology. At a minimum, patients expected the cause or diagnosis of disease, the seriousness of the illness, how they should take the medicines, and the preventive aspects of the disease. It was a common complaint of the patients that the physicians did not explain sufficiently, with some rare exceptions of course. Physicians were divided in their opinion regarding the importance of giving explanations to patients. Some physicians said it was the right of the patients to receive an explanation of their health condition. Some said explanation was important only in critical diseases like liver cirrhosis, tuberculosis, etc., while others stated giving an explanation to patients was useless unless the patient was educated, conscious, and willing to receive an explanation (Table 3).

When asked whether patients need to be involved in care-related decision-making, patient respondents expressed unfamiliarity with this notion and expressed that the physician should decide what is best for the patients. A patient respondent, who was the caregiver of her elderly mother suffering from diabetes and eye conditions, however, requested during the IDI to involve the patients in providing care to their sick family members, especially in chronic conditions. Both patients and physicians confirmed the nonexistence of a formal way of getting patients or their family members into the treatment process. In observations too, we did not find any patient to be involved in therapeutic decision-making or care giving.

Preventive issues, especially diets, are traditionally seen as integral to Bengali therapeutic culture. Therapeutic diets are known as *'pottho'* and are always pronounced in the same phrase with medicine, like *'oshudh-pottho'*, meaning 'medicine and diet'. Patients expected that the physician, along with the treatment of the disease, would also explain in details about diet, foods that are allowed or forbidden, and prevention of the disease. In observations, we found that physicians usually explained disease prevention and health promotion measures related to the disease of the patient (Table 3), but did not give general health promotion advice.

Many patients, especially in FGDs with females, shared their stories on what difficulties they faced in following prescriptions due to physicians’ bad handwriting. One female FGD respondent shared the following story:

*"A doctor prescribed me a drug when I was pregnant*. *The doctor prescribed a drug related to my pregnancy condition; but the medicine salesman in the dispensary mistakenly gave some medicine for asthma*, *as they could not read doctor's handwriting*. *For some reasons*, *I was suspicious that this might be a wrong medication*. *So*, *I did not take the medicine and went to the doctor again*. *Seeing the medicine the doctor was shocked and said if I had that medicine it would be terribly harmful to my fetus*. *So*, *it’s important for doctor's handwriting to be clear*.*"* [FGD participant, female]

#### Gaining trust

Patients did not express great concern over confidentiality. One physician said, “*Patients in our country*, *contrary to western notions*, *rather preferred their information to be shared*.” He gave the example of the nationwide surveillance against polio eradication campaign. Physicians had to share the patients' information with several national and international agencies, and patients were appreciative of this. They supposedly thought they would get better attention and treatment if their information had been shared with different stakeholders (Table 3). Physicians mentioned it was important to keep some sensitive information secret, in congruence with the professional codes of ethics. In observations, we found no patient information was preserved properly, so leaking of information was out of the question. However, the NGO-clinic had a specific protocol to maintain the confidentiality of patients' data.

Patients said they expected that the physician would refer the patient immediately. Patients alleged that physicians seldom referred their patients because,

*"*…*they don't want to let their patients go*, *this is their mentality*. *They think*, *'if I let this patient go to a different doctor*, *and if the patient gets cured*, *that doctor will earn name and my business will be ruined*.*‴* [IDI with a teacher, female, 45 years]

Physicians denied these allegations, saying they were quick to refer; it was the patients who often did not comply for fear of the hassle and cost. In observations, we found there was hardly any instance of referral in the public sector, as physicians perceived poor patients could not afford to go to higher-level health facilities. Interestingly, we found physicians in private clinics referred patients more readily (Table 3). When probed about this, physicians replied their patients were wealthier and the physicians and the private clinics wanted to stay away from the troubles of complicated patients.

Contrary to patients’ expectations, physicians often avoided seeking assistance from other colleagues when they experienced confusion. On one occasion, a pregnant woman came to the NGO-clinic, and the physician performed ultra-sonogram of the abdomen. It was presumed from the observation that the physician was not confident about the findings. After looking at the printout of the ultra-sonogram for several minutes, the physician told the patient that everything was normal. His lack of confidence was evident from his voice and appearance. The patient then stated that another physician told her she was having twins. Then the physician took back the ultra-sonogram, looked at it again, and said it indeed was a twin pregnancy. In another occasion, in a public sector setting, we observed a young physician failing to diagnose a skin condition. The observer suspected this from his own clinical training and experience. The physician prescribed a drug and the patient left. After the patient’s departure, the observer asked the physician whether he was sure of the diagnosis. The physician admitted that he was not. We also observed a physician's inability to understand an X-ray, electrocardiogram, and diagnose other diseases. There were other physicians nearby, but the physician neither asked them nor informed the patient of her/his inability to make a clear diagnosis (Table 3).

Patients demanded the physicians should not say or do anything that might breach their trust. Examples of such trust-shattering behavior included: telling the patient to do a test from any specific diagnostic center, encouraging them to buy medicines from a specific pharmaceutical company, asking the patient (in public sector) to come visit him in a private clinic, and moonlighting. When asked how a physician might earn trust, patients emphasized physicians' providing sufficient explanations for the treatments and tests.

Patients also complained that physicians have become more business-oriented rather than service- or care-oriented. One example of businesslike behavior of physicians was demanding fees forcibly from incapable patients. Patients expressed the highest distaste for physicians' suggesting patients do diagnostic tests from a specific diagnostic center. They alleged that, although some physicians did not do this, some even forced the patients to do so (Table 3).

Patients did not want to see the physicians involved in illegal or unethical activities such as: taking money from patients against free services (public sector), bringing patients in their own private clinics with the help of *dalals*, collusion with diagnostic centers, accepting gifts from pharmaceutical representatives and prescribing substandard medicine, and taking advantage from *dalals* in various ways. In observations, however, we did not find any physician visibly involved in such illegal or unethical activities; but some activities might seem to have tacit approval. For example, physicians in the public sector allowed *dalals* around the chamber, or they did not take any steps to expel them. In a private clinic, we saw a *dalal* receiving money in exchange for enticing a patient away from another clinic (Table 3).

#### Optimizing benefits

Based on his experiences of violence against his female patients, a senior public sector physician suggested a physician should talk with the perpetrator if needed and report such incidents immediately to the concerned authority (Table 3). The physicians should solve the family problems of patients if it is related to their health condition (e.g., torturing by husband, family feud, etc.) with the help of the concerned person of that area (e.g., political representative, administrative personnel, health sector personnel, etc.).

Home visits by physicians were culturally expected from the patients. In interviews with formal sector physicians, we realized they were reluctant to visit patients at home (Table 3), leaving this practice mostly for the village doctors. Village doctors usually went for home visits, but they were also often reluctant on the ground of financial issues. We found the most elderly private sector physician (retired from the public sector) still going for home visits, but he did it reluctantly only when the patient earnestly requested it.

When probed whether a physician should be considerate of individual needs of a patient, patient respondents expressed that the physician should indeed provide treatment complying with patients’ individual needs. One patient shared his story, which is as follows:

*"One of my sons had an accident once*. *I took him to Dr*. *(X)*. *He suggested me to take my son to Dhaka to check if he had a fracture on his skull*. *I said*, *my other son has an exam now*, *how can I leave that son here*? *I need to take that son to the exam location*, *which is far from here and he cannot go alone*. *The doctor then checked my son again and started treating himself*. *He is cured now*, *and I did not have to hamper my other son's education too*.*"* [FGD participant, male]

We observed that a physician strived to facilitate the services at the locality of the patient, which was an instance of responsiveness. The excerpt from observation notes in this regard is as follows:

*"I saw that the doctor prescribed saline to a woman*. *He was asking the patient how she would administer the saline at home*. *The patient said that a village doctor near her home may help*. *Doctor asked the name of the village doctor*. *Patient mentioned the name*. *The doctor said he had heard his name and he is reliable*. *She can go and get the saline pushed by the village doctor*.*"* [Private sector observation note, 7th day of observation, 11:30 am]

Patients demanded in IDIs and FGDs that the physicians consider the financial strength of the patients and help them get treatment within their financial ability. Helping the needy patients, according to them, may involve the following steps: understanding the financial condition of the patient, giving ideas about treatment cost, and helping the patient if necessary. When physicians were asked if and how they tried to understand the financial condition of the patients, they replied they often did this, especially in the public sector, by asking the patients directly about their income or by asking him indirectly (such as asking his profession). They said patients themselves also commonly expressed their inabilities. Besides these, according to physicians, it might be guessed by observing the patient’s conversation, behavior, and clothing. In terms of giving an idea of treatment costs, physicians in both public and private sectors denied this was their responsibility (Table 3), saying it was the job of pharmacists or medicine retailers. Village doctors, conversely, were quite good at this, which they attributed in the IDIs to the fact that most of them ran medicine shops alongside their consulting patients. In observations, we found some instances of helping the needy patients by physicians. These included prescribing low-cost antibiotics, taking a lower or no consultation fee (in case of private physicians), helping patients from a ‘poor fund’ (a fund often organized by a group of physicians), helping to get free medicines from the hospital (in case of government physicians), focusing on the history and physical examination to avoid investigation, prescribing the essential tests only, compromising the commission paid by diagnostic centers to the physician for each test, and recommending a treatment method that saves money (Table 3).

## Discussion

This study explored the responsiveness of physicians and grouped the components of responsiveness under five domains: friendliness, respecting, informing and guiding, gaining trust, and optimizing benefits, using qualitative methods from a District in south-west part of Bangladesh. Few themes cut across the five domains of responsiveness of physicians; these are–the gaps between physicians’ and patients’ perceptions and practices regarding responsiveness, importance of context in understanding responsiveness, and few aspects of responsiveness where physicians lived up to the expectations of service seekers. These crosscutting themes are presented in Table 3.

Most of these overarching themes have congruence with relevant studies from similar settings. For example, the difference between physicians’ and patients’ perceptions and practices regarding many of the reported responsiveness domains (viz., hierarchy, not providing explanation, not allowing questions, lack of financial sensitivity) has also been described by Zaman [26] in an ethnographic study of a hospital in Bangladesh. That study suggested that this difference may have stemmed from the different backgrounds, education, and social position of physicians and patients. Kleinman [42] described consultations as a transaction between the lay (i.e., patients’) and professional (i.e., physicians’) explanatory models, where this transaction is influenced by their difference in power. This power differential may be based on social class, ethnicity, age, gender, or other sociodemographic factors. Helman [43], on the other hand, argues that even if physicians and patients come from similar backgrounds, their perspectives on the interaction between them would still be different. This difference is due to their different premises, such as employment of different systems of proof, assessment of efficacy of treatment in different ways. He gave examples of how the presentation of the same illness can be seen very differently from the perspectives of the physician and the patient.

This study identified differences between Bangladesh and other countries, especially Western settings, in terms of perceptions and practices around responsiveness (Table 3). Roter and Hall [39] described four types of interactions between physician and patients, in terms of degree of control. When physician has greater control over therapeutic decision-making, it is called *paternalistic;* when there is a balance, it is *mutual;* a *default* is when neither has a clear control; and finally, a *consumerist* approach is when patient has the larger control. In Bangladesh, patients preferred a *paternalistic* consultation interaction, while, according to a study on European patient’s views on responsiveness of health providers, most patients preferred *interactive* (51%), and some even *consumerist* (23%) approach [33]. Similar discrepancy was found in terms of autonomy of patients in selecting physicians too. Understanding of these context-specific issues can aid in understanding some related debates, e.g. allowing autonomy to choose providers, shared decision-making in treatment, degree of confidentiality of information, disclosure of financial status of patients before prescribing, importance of consent in therapeutic setting, etc. Understanding of context is also important in training of physicians, as the clinical methods textbooks can draw examples from them and provide context-specific education. Thus, context in responsiveness is important as this informs the positionality from which people view the elements of responsiveness.

Physicians, despite shortcomings, often demonstrated responsiveness, even in the face of absent or inadequate health system support. The limitations within which physicians provide services in Bangladesh are documented in various publications [30,37,44,45]. Our finding that physicians reassured many patients is supported by another study, where the rating by patients regarding reassurance provided by physicians was better among other service related indicators [27]. Our finding regarding the tendency of physicians to financially assist the poor patients is also supported by the findings from Zaman’s [26] ethnographic study.

### Strengths and limitations of the study

One of the strengths of this study was that the first author stayed for a prolonged time of three months (July–September, 2014) in the field: contacting the gatekeepers, developing a list of respondents, obtaining permission, building rapport with the respondents as well as the community, and formal data collection. Secondly, we employed different methods (IDI, FGD, observation) and data sources (physicians, village doctors, service users) for triangulation. Transferability and dependability of the work were ensured by providing thick description of the research process, and by consulting with experts, respectively. For confirmability, audit trail of all the steps were recorded; raw data, analytic products, and research instruments were saved systematically.

A limitation of this study is the power relationship and hierarchical distance between the researcher and the subjects. Local constructs around gender, class, language, and age might come into play during the interaction between the researcher and the respondents, which might consequently affect the quality of the data. The Hawthorne effect might restrict the observer from observing the real behaviors of physicians [46–48]; a longer duration of observation might be more appropriate to avoid this limitation. Thirdly, owing to sampling the female FGD participants from educational institutions, the level of education of the respondents were higher than the other Bangladeshi rural women. Therefore, their experience of interaction with physicians, and expectations from them would be different than general female population.

### Suggestions for future studies

This study was conducted in rural areas, where most of the service seekers belonged to a lower socio-economic and educational group. Perceptions around responsiveness may be different in an urban setting. This study was conducted in an outpatient setting, so understanding of responsiveness in other settings, such as inpatient, emergency, delivery ward, and maternity care may be useful too.

A quantitative survey, developed based on the qualitative findings, may allow us to measure the status of responsiveness in comparable settings. A psychometric study including factor analysis may allow us to examine the dimensionality of the domains. A scale of responsiveness can be developed to measure responsiveness in rural Bangladesh, and can be validated in relevant settings.

Responsiveness of physicians may be influenced by certain factors, most notably training and health systems support. In order to improve the responsiveness of physicians, first it would be helpful to know about the determinants of responsiveness or the factors rendering physicians unresponsive.

### Policy implications

Responsiveness has been gaining attention in the international policy context. The World Health Organization’s framework for HRH performance included responsiveness as its one the four components [3]. This requires contextualization in terms of policy development. The specificity of the context (and departure from established Western notions) guided by local knowledge regarding responsiveness is crucial.

Once further research clarifies the nature of responsiveness across social groups and health care settings, policy measures can be developed for provider training and assessment. Research on responsiveness can inform reforms in the medical curriculum that integrate the social expectations of the patients during care seeking pathway. This can be further strengthened through practical experiences during the internship-training period and beyond. Effectiveness of such trainings have been demonstrated in several studies and systematic reviews [49,50]. Subsequent to training, introducing responsiveness as one of the indicator for performance could improve the physician’s approach and behavior towards the patients.
